# Comparative Efficacy of Monobutyrin, Tributyrin, Sodium Butyrate, and Poly-β-hydroxybutyrate on Growth, Intestinal Health, and Nitrite Stress Resistance in *Penaeus monodon*

**DOI:** 10.3390/antiox15080929

**Published:** 2026-07-27

**Authors:** Yafei Duan, Ruijie Zhu, Yun Wang, Jianhua Huang, Song Jiang, Qibin Yang, Yundong Li, Jianzhi Shi, Yukai Yang, Lishi Yang, Yangyang Ding, Falin Zhou

**Affiliations:** 1 State Key Laboratory of Mariculture Biobreeding and Sustainable Goods, Key Laboratory of South China Sea Fishery Resources Exploitation & Utilization, Ministry of Agriculture and Rural Affairs, South China Sea Fisheries Research Institute, Chinese Academy of Fishery Sciences, Guangzhou 510300, China; duanyafei@scsfri.ac.cn (Y.D.);; 2 Key Laboratory of Efficient Utilization and Processing of Marine Fishery Resources of Hainan Province, Sanya Tropical Fisheries Research Institute, Sanya 572018, China; 3 Shenzhen Base of South China Sea Fisheries Research Institute, Chinese Academy of Fishery Sciences, Shenzhen 518121, China; 4 Southern Marine Science and Engineering Guangdong Laboratory (Zhuhai), Zhuhai 519080, China

**Keywords:** shrimp, Nrf2 signaling, antimicrobial peptides, short-chain fatty acids (SCFAs), microbial community, weight gain, growth

## Abstract

Intestinal health is crucial for the growth and stress resistance of shrimp. Butyrate, a beneficial metabolite of intestinal microbiota and the primary energy substrate for enterocytes, exerts regulatory effects on intestinal health. Butyrates exist in various chemical forms, yet their application in shrimp remains limited. Therefore, in this study, *Penaeus monodon* were fed diets supplemented with 1% four types of butyrate (monobutyrin, MB; tributyrin, TB; sodium butyrate, SB; poly-β-hydroxybutyrate, PHB) for 56 days, followed by 48 h of acute nitrite stress. A systematic investigation into their influences on the shrimp growth, intestinal health and nitrite stress resistance was conducted. The results showed that the four butyrate types significantly increased the weight gain rate of the shrimp by more than 25% and improved the survival rate under nitrite stress by more than 28% when compared with the control group (*p* < 0.05). They also improved intestinal mucosal integrity, and enhanced intestinal antioxidant and immune capacities through the activation of the *Nrf2* pathway and the upregulation of immune gene expression. Specifically, T-AOC and SOD activities, as well as the expression levels of *Nrf2*, *GPx*, *Trx*, *ALF*, *Pen3*, and *serP* genes, were significantly upregulated in all four butyrate groups, while MDA content was significantly decreased (*p* < 0.05). In addition, LPO content, CAT and ASC activities, and the expression of *HO1*, *SOD*, *Crus*, and *proPO* genes exhibited differential changes among the four butyrate groups. Furthermore, the intestinal microflora structure was reshaped by all four butyrate variants, with notable reductions in pathogenic *Vibrio* alongside elevated abundances of advantageous taxa including Rhodobacteraceae. In conclusion, butyrate can facilitate the growth and anti-stress capacity of *P. monodon* by improving intestinal health, with the overall efficacy ranked as TB, PHB, MB and SB under the present study conditions.

## 1. Introduction

The black tiger shrimp *Penaeus monodon* ranks among the world’s most economically vital cultured shrimp varieties [1]. The rapid expansion of intensive farming systems has unavoidably led to the deterioration of water quality and the recurrent occurrence of environmental stress, which greatly hinders stable production and economic returns in shrimp farming [2]. Among various stressors, nitrite is a prevalent and hazardous environmental stressor in shrimp aquaculture. Nitrite is a toxic intermediate produced during aquatic nitrogen metabolism, primarily resulting from the accumulation of residual feed and shrimp excreta, as well as the imbalance of microbial communities in the culture water [3]. Nitrite can induce stress responses in shrimp, leading to systemic metabolic disorders, immunosuppression, increased disease susceptibility, and intestinal damage, ultimately compromising shrimp growth and survival [4,5,6,7,8]. Accordingly, the development of eco-friendly strategies to alleviate nitrite stress is essential for promoting healthy and sustainable shrimp aquaculture.

The intestine acts as a pivotal organ governing nutrient digestion and absorption, immune defense, and internal homeostasis in shrimp. The structural and functional integrity of the intestines fundamentally determines shrimp growth performance and anti-stress capacity [9]. Resident intestinal microbes regulate host nutritional metabolism and immune homeostasis; dysbiosis of microbiota readily damages the intestinal barrier, triggering intestinal lesions and weakened disease resistance [10,11,12]. Short-chain fatty acids (SCFAs) are saturated fatty acids with carbon chain lengths of ≤6, mainly including acetate, propionate, and butyrate, which are produced by the fermentation of dietary fiber and other carbohydrates by the host intestinal microbiota [13,14]. SCFAs can sustain intestinal health via multiple mechanisms including providing energy for intestinal epithelial cells, repairing mucosal injury, optimizing microbial composition, and activating antioxidant and immune-related signaling pathways [13,14]. Accordingly, SCFAs function as core regulatory mediators linking intestinal microecology and host physiological health.

As a vital member of SCFAs, butyrate is a four-carbon fatty acid with the molecular formula C_4_H_8_O_2_. It acts as the major energy source fueling intestinal epithelial cells, and represents a promising green feed additive with great application potential in aquaculture [15,16,17]. Currently, the commonly used butyrate derivatives in shrimp aquaculture mainly include monobutyrin (MB), tributyrin (TB), sodium butyrate (SB), and poly-β-hydroxybutyrate (PHB). For example, dietary MB can improve the growth, feed efficiency, digestion, innate immunity, and antioxidant capacity in *Litopenaeus vannamei* [18]. TB can enhance the growth and intestinal health of *L. vannamei* by enhancing antioxidant and immune capacities and reducing inflammation [19,20,21,22]. PHB can promote the growth and intestinal health of *L. vannamei* [23,24,25]; it can also improve the immune response and resistance to *Vibrio parahaemolyticus* infection in *L. vannamei* [26]. SB can promote the growth of *L. vannamei* [27,28,29,30,31]; improve hemolymph immunity, antioxidant capacity, and stress resistance in *L. vannamei* [28]; and inhibit *V. parahaemolyticus* and decrease the occurrence of vibriosis in *L. vannamei* [30,31]. Most existing butyrate studies in shrimp have focused on *L. vannamei*, yet the marked species-specific differences in physiology, feeding, and culture prevent direct extrapolation of these findings to *P. monodon* farming.

Notably, different types of butyrates possess distinct physicochemical properties, which result in differences in their release patterns, stability, efficacy, and bioavailability within the gastrointestinal tract [32]. For instance, SB is water-soluble and rapidly releases butyrate in the stomach and upper intestine, leading to uneven butyrate delivery along the intestine [32]. MB and TB are esters characterized by gastric bypass and sustained intestinal release, with the distinction that TB liberates more butyrate than MB [18]. PHB is a high-molecular-weight polyester that can be degraded by intestinal bacteria, offering enhanced sustained-release properties while also functioning as a carbon source to directionally modulate the intestinal microbiota [23,24]. Research has further demonstrated that TB exhibits stronger and more direct cellular effects and superior pharmacokinetic profiles compared with SB [33], and is more effective in promoting growth and digestion in *L. vannamei* [34]. However, at present, it remains unclear whether MB, TB, SB, and PHB exhibit differential effects on shrimp growth, intestinal health, and stress resistance. This uncertainty hinders the targeted selection of butyrate types in shrimp aquaculture and thus warrants in-depth investigation.

Therefore, in the present study, four forms of butyrate (MB, TB, SB, and PHB) were supplemented in *P. monodon* diets to separately explore their impacts on growth, nitrite tolerance, and intestinal health of shrimp. The main research objectives included: (1) growth performance and feed utilization; (2) survival rate and resistance to nitrite stress; (3) intestinal mucosal histomorphology; (4) intestinal antioxidant capacity; (5) intestinal immune parameters; and (6) intestinal microbial community. These findings will help elucidate the regulatory roles of dietary butyrates on intestinal health in *P. monodon*, identify the optimal form of the four forms of butyrate, and provide theoretical support for their application as feed additives.

## 2. Materials and Methods

### 2.1. Shrimp Feed Formulation Procedures

The MB and TB were supplied by Guangzhou Bioroad Biotechnology Co., Ltd. (Guangzhou, China). The SB was obtained from Shanghai Yuanye Bio-Technology Co., Ltd. (Shanghai, China). The PHB was supplied by Ningbo Tianan Biological Material Co., Ltd. (Ningbo, China). Based on the basal diet for *P. monodon*, five experimental diets were formulated. The basal diet served as the control group (CK) without any butyrate supplementation. Four experimental diets were separately supplemented with MB, TB, SB and PHB at an equal dosage of 1% diet, respectively. This supplementation level was primarily based on previous reports in *L. vannamei* [16,17,23], given that *P. monodon* shares similar feeding habits and digestive physiological characteristics with *L. vannamei*. The ingredient formulas and nutritional profiles of test feeds are listed in Table S1, all of which were manufactured following the protocol reported by Wang et al. [35]. Proximate composition analysis confirmed no obvious differences in basic nutritional components among the five formulated diets.

### 2.2. Shrimp and Rearing Conditions

The *P. monodon* were obtained from the indoor ponds at the Shenzhen Base of South China Sea Fisheries Research Institute, which exhibited uniform healthy status and average body weight of 2.09 ± 0.02 g. Before the formal feeding experiment, the shrimp were domestically raised for seven days in 400 L seawater test tanks. The seawater was fresh, filtered, and continuously aerated, with a salinity of 30, pH of 8.2–8.4, temperature 30 ± 0.5 °C, and dissolved oxygen (DO) concentration above 6.0 mg/L. Throughout the domestication period, the shrimp received basal pellet feed at a daily ration equivalent to 5% of their body mass. Daily water exchange of two-thirds volume was performed, and leftover feed as well as excreta were cleared timely to maintain stable, safe water conditions.

### 2.3. Feeding Trial and Sample Collection

After acclimation, shrimp were randomly assigned to five groups: a control (CK) and four experimental groups (MB, TB, SB, and PHB), each receiving the respective diet. Each group comprised three replicate tanks, with 40 shrimp per tank. A fine-mesh cover was placed over each tank to prevent shrimp from jumping out and to avoid external disturbances. Shrimp were supplied feed daily at a ration accounting for 5% of their total body mass, with feedings administered three times a day (07:00, 12:00, and 18:00). The experiment was conducted in an indoor workshop under a natural photoperiod. Residual feed pellets were collected, dried, and weighed 1–1.5 h after each feeding. During the experimental period, the shrimp rearing management and water quality parameters were identical to the conditions maintained throughout acclimation. The whole feeding experiment spanned a period of 56 days.

After a 56-day feeding trial, intestinal tissues were randomly sampled from shrimp individuals in every tank. Specifically, three intestines per tank were harvested for histological analysis; three intestines per tank were collected for biochemical indicators analysis; three intestines were stripped of feces, pooled and preserved in RNAFollow reagent for gene expression quantification; and three intestines with intact feces were combined for microbiota profiling.

### 2.4. Nitrite Stress Exposure Test

Upon completing the 56-day feeding experiment, a 48 h nitrite stress challenge test was implemented. Briefly, 10 shrimp individuals were randomly picked from each replicate tank of every treatment for acute nitrite exposure. The target nitrite-N concentration was set to 20 mg/L via supplementing sodium nitrite into freshly filtered seawater. Water nitrite concentrations were determined every 4 h throughout the trial to maintain stable exposure levels. Nitrite concentration in the rearing water was measured by the N-(1-naphthyl)-ethylenediamine dihydrochloride spectrophotometric method. Rearing conditions remained consistent with those applied during the preceding feeding experiment. Following the 48 h stress exposure, the shrimp survival rates within each tank were documented.

### 2.5. Growth Performance Analysis

Upon completion of the 56-day feeding experiment, the shrimp quantities and individual body weights in each replicate tank were measured. Growth indices and feed efficiency indicators were computed using the following equations:

Weight gain (WG, %) = 100 × (final body weight − initial body weight)/initial body weight

Feed conversion ratio (FCR) = dry feed intake/(final body weight − initial body weight)

Survival rate (SR, %) = 100 × (final shrimp number/initial shrimp number)

### 2.6. Histomorphological Analysis

Intestinal specimens were immersed in 4% paraformaldehyde for 24 h to complete fixation. After 30 min of running water washing, the specimens underwent gradient ethanol dehydration, xylene transparency and paraffin embedding sequentially. Continuous slices with a thickness of 4 μm were sectioned via a microtome (Leica RM2016, Shanghai, China). After hematoxylin-eosin (H&E) staining, all slices were observed under an optical microscope (Nikon, Tokyo, Japan). Detailed experimental procedures are provided in the Supplementary Materials.

### 2.7. Biochemical Analysis

Sterile normal saline was utilized to homogenize intestinal tissues and formulate 10% tissue homogenates. The obtained homogenates were subsequently centrifuged at 4 °C with a rotational speed of 3500 rpm over a 15 min duration. The resulting supernatants were harvested to determine biochemical markers associated with oxidative stress. Among these, malondialdehyde (MDA) and lipid peroxidation (LPO) represent oxidative stress damage, while total antioxidant capacity (T-AOC), anti-superoxide anion capacity (ASC), total superoxide dismutase (SOD), and catalase (CAT) reflect antioxidant capacity. All the indicators were measured using commercial kits (Nanjing Jiancheng Bioengineering Institute, Nanjing, China) from the same batch, and the final detection was performed on a microplate reader (Spark, Tecan, Grödig, Austria). Detailed experimental procedures are provided in the Supplementary Materials.

### 2.8. mRNA Expression Level Analysis

TRIzol^®^ reagent (Invitrogen, Carlsbad, CA, USA) was adopted to extract total RNA from shrimp intestinal tissues. Once the integrity and purity of extracted RNA were verified, complementary DNA (cDNA) was synthesized via reverse transcription with the Servicebio^®^ RT First Strand cDNA Synthesis Kit (Servicebio, Wuhan, China). Quantitative real-time PCR (qPCR) reactions were conducted on a Heal Force CG-02 platform utilizing the SYBR Green Premix *Pro Taq* HS qPCR kit (Accurate Biotechnology (Hunan) Co., Ltd., Changsha, Hunan, China). The *P. monodon* β-actin gene served as the housekeeping reference gene for normalization. All oligonucleotide primer sequences applied in qPCR assays are summarized in Table S2. The Livak–Schmittgen calculation approach [36] was adopted to quantify relative transcript abundances of target genes, with expression data exhibited as fold variations compared against the CK group.

### 2.9. Intestinal Microbial Community Analysis

Total genomic DNA of intestinal bacteria was extracted via the FastDNA SPIN Kit (MP Biomedicals, Santa Ana, CA, USA). The integrity of extracted genomic DNA was validated with 1% agarose gel electrophoresis, followed by measurements of DNA purity and concentration. After passing quality assessment, the V4 hypervariable region of bacterial 16S rRNA gene was amplified with the primer set 515F/806R. All PCR reactions were carried out utilizing TransStart Fastpfu DNA Polymerase. Amplified PCR amplicons were checked on 2% agarose gels and then purified with the AxyPrep DNA Gel Extraction Kit (Axygen Biosciences, Union City, CA, USA). Next, DNA quantification was completed on the QuantiFluor^TM^-ST Blue Fluorescent Quantitation System (Promega, Madison, WI, USA). Qualified PCR products were blended at matching ratios in line with individual sample sequencing demands. Sequencing libraries were built using the NEBNext^®^ Ultra™ DNA Library Prep Kit for Illumina (New England Biolabs, Ipswich, MA, USA), and paired-end 250 bp sequencing was implemented on the Illumina PE250 platform.

Raw sequencing reads were preprocessed with FLASH software to screen valid sequences relying on barcode labels. Trimmomatic and Usearch tools were utilized to conduct quality trimming and filtration on raw data. Chimeric sequences were detected and eliminated through the UCHIME algorithm. Operational taxonomic units (OTUs) were clustered at a 97% nucleotide similarity cutoff by Usearch v10 software. Taxonomic annotation for representative OTU sequences was accomplished via the RDP Classifier (version 2.2) against the Silva 132 reference database, with a confidence threshold set to 0.7. Relative abundance profiles of intestinal bacteria were analyzed at both phylum and genus taxonomic ranks. Mothur v1.30.1 was employed to calculate alpha diversity metrics including Chao1, Simpson, and Shannon indices. R programming language was applied to generate and interpret OTU distribution Venn plots as well as principal component analysis (PCA) for beta diversity comparison. Linear discriminant analysis effect size (LEfSe v1.0) was deployed to identify biomarker bacterial taxa with significant intergroup differences. PICRUSt v2.6.2 software was used to predict intestinal microbial metabolic capacities corresponding to Level 3 KEGG pathways, and the RandomForest v4.6-14 package in R was adopted for subsequent functional profiling. Key differential indicators were screened using a random forest model, with variable contributions evaluated by the Gini coefficient and significance assessed via permutation test (*p* < 0.05).

### 2.10. Statistical Analysis

All the experimental outcomes, including growth, survival, biochemical, and gene expression data, were expressed as mean values accompanied by standard error (SE). Each group included three biological replicates. Normality distribution and variance homogeneity of all datasets were separately evaluated via Shapiro–Wilk test and Levene’s test at first. Afterwards, one-way ANOVA was implemented, and Tukey’s multiple comparison test was adopted for post hoc analysis. The whole statistical computations were completed with SPSS 21.0 software. Statistical significance was defined as *p*-value less than 0.05.

The above data were tested for normality using the Shapiro–Wilk test, which indicated small within-group variability, no significant outliers, and approximate normal distribution. Levene’s test confirmed homogeneity of variances among groups, fulfilling the assumptions for ANOVA.

## 3. Result

### 3.1. Growth Performance of the Shrimp

**Figure 1:**
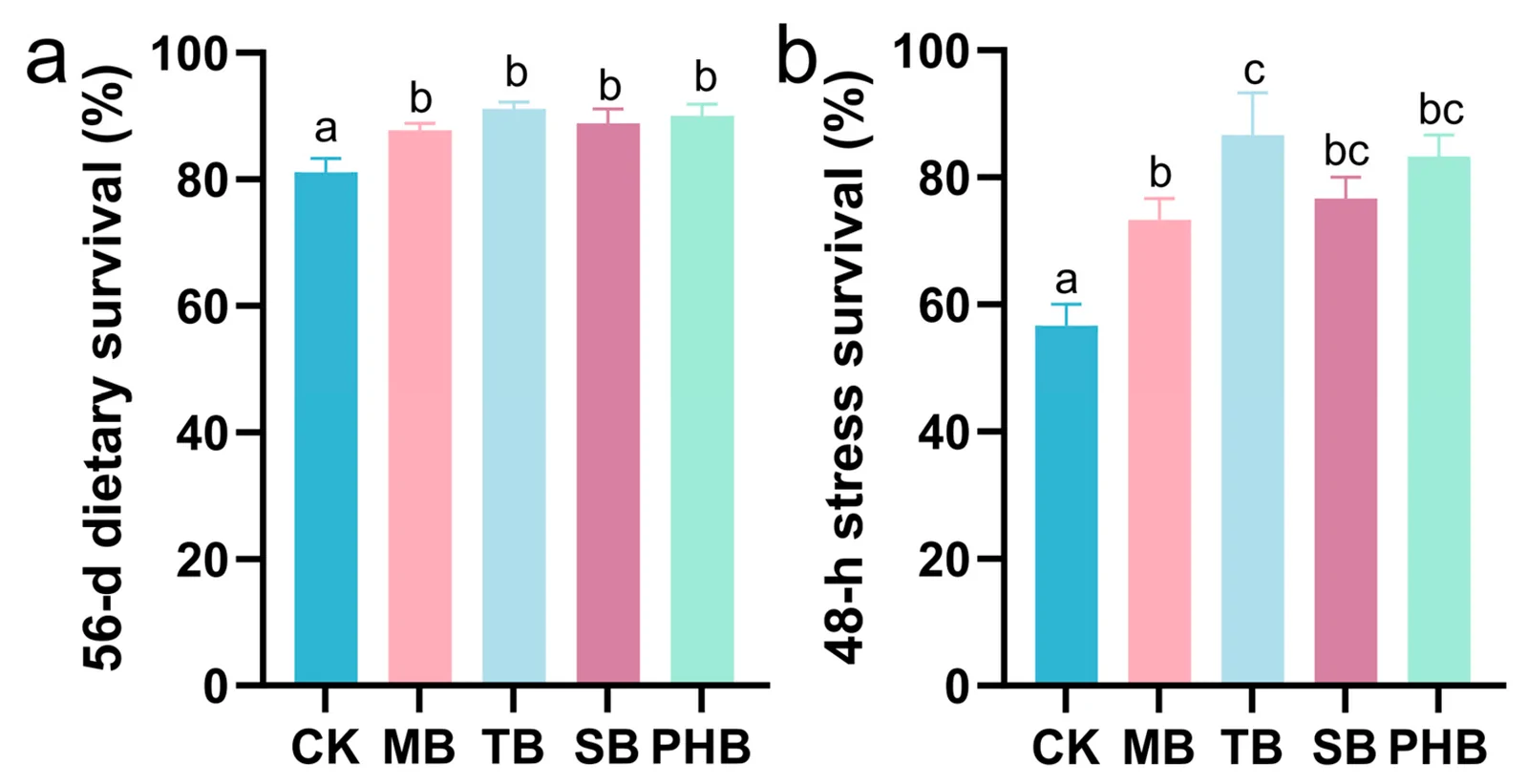
The survival of *P. monodon* fed diets supplemented with different types of butyrates for 56 days and subsequent 48 h nitrite stress. (**a**) Survival rate after 56 days of butyrate feeding; (**b**) survival rate under 48 h of 20 mg/L nitrite stress after 56 days of butyrate feeding. Different lowercase letters above error bars indicate significant differences among groups (*p* < 0.05).

The initial body weight of shrimp did not differ significantly among the five groups, suggesting that the baseline conditions were comparable and thus could not confound the comparison of growth performance. After a 56-day feeding trial with diets containing different types of butyrates, the four supplemented groups had significantly higher final weight and weight gain than the CK group (*p* < 0.05). Among them, the weight gain values in the TB and PHB groups were the highest, while those in the MB and SB groups were lower than those in the TB and PHB groups. The FCR was markedly lower in the four supplemented groups than the CK group (*p* < 0.05), with the lowest values found in the TB and PHB groups, then the MB and SB groups (Table 1). Moreover, the survival rates of the four supplemented groups were significantly elevated relative to the CK group (*p* < 0.05), whereas the four groups did not differ significantly from one another (*p* > 0.05) (Figure 1a). Following 48 h of nitrite stress, dietary butyrate supplementation significantly improved the shrimp survival (*p* < 0.05), and the TB group had the highest survival, with the PHB group ranking second (Figure 1b).

### 3.2. Intestinal Histomorphological Changes

**Figure 2:**
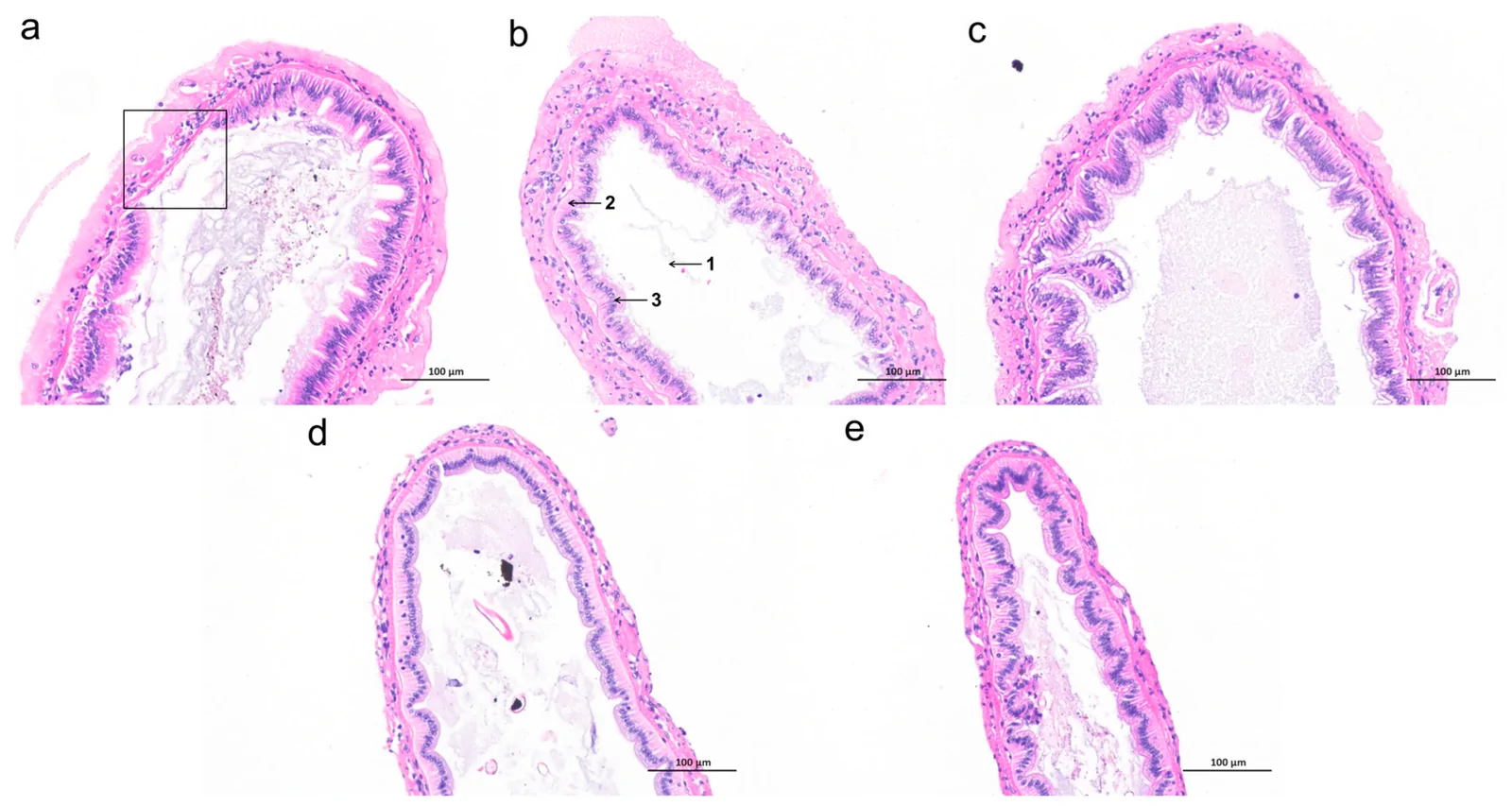
The histomorphological observation of *P. monodon* fed diets supplemented with different types of butyrates for 56 days. (**a**) CK group; (**b**) MB group; (**c**) TB group; (**d**) SB group; (**e**) PHB group. ×200. 1, mucosal epithelium; 2, brush border; 3, nuclei. The box indicates the damaged intestinal mucosa.

The integrity of the intestinal mucosa was analyzed following the dietary addition of four butyrate formulations. The intestinal mucosa of the CK group exhibited poor integrity with loosely arranged epithelial cells, and obvious mucosal exfoliation was observed in some areas (Figure 2a). In contrast, all four butyrate-supplemented groups showed varying degrees of improvement in intestinal mucosal morphology, characterized by good integrity of the mucosal structure without obvious mucosal shedding, and more compact and orderly arrangement of epithelial cells (Figure 2b–e).

### 3.3. Intestinal Oxidative Stress-Related Biochemical Indicators Changes

**Figure 3:**
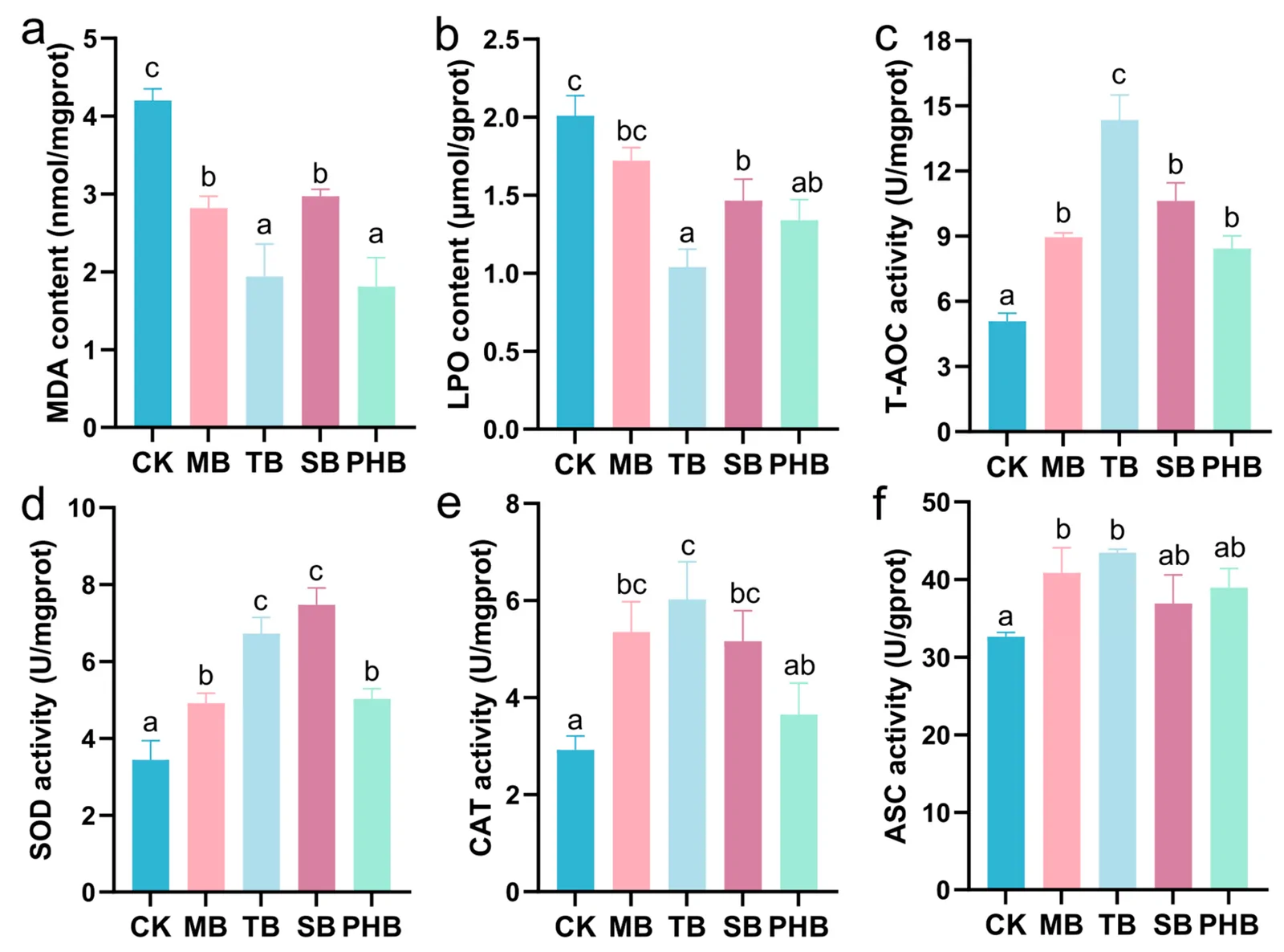
The changes in oxidative stress-related biochemical indicators in the intestine of *P. monodon* fed diets supplemented with different types of butyrates for 56 days. (**a**) MDA content; (**b**) LPO content; (**c**) T-AOC activity; (**d**) SOD activity; (**e**) CAT activity; (**f**) ASC activity. Different lowercase letters above error bars indicate significant differences among groups (*p* < 0.05).

The changes in oxidative stress-related biochemical parameters in the shrimp intestines were investigated after dietary supplementation with different types of butyrates (Figure 3). Compared with the CK group, the four supplementation groups showed a significant reduction in MDA content (*p* < 0.05). Meanwhile, LPO levels were also significantly lower in the TB, SB, and PHB groups (*p* < 0.05), but slightly reduced without significance in the MB group (*p* > 0.05). Among them, MDA in the TB and PHB groups possessed remarkably reduced concentrations in comparison with the MB and SB groups (*p* < 0.05); LPO content was lowest in the TB group. In addition, antioxidant indicators such as T-AOC and SOD activities were significantly elevated in the four supplementation groups (*p* < 0.05); CAT activity was significantly increased in the MB, TB and SB groups (*p* < 0.05), but slightly increased without significance in the PHB group (*p* > 0.05); and ASC activity was significantly increased in the MB and TB groups (*p* < 0.05), but slightly increased without significance in the SB and PHB groups (*p* > 0.05). Among them, T-AOC and CAT activities reached their maxima in the TB group; SOD activity was maximal in the TB and SB groups; and ASC activity was maximal in the MB and TB groups.

### 3.4. Intestinal Antioxidant-Related Gene Expression Changes

**Figure 4:**
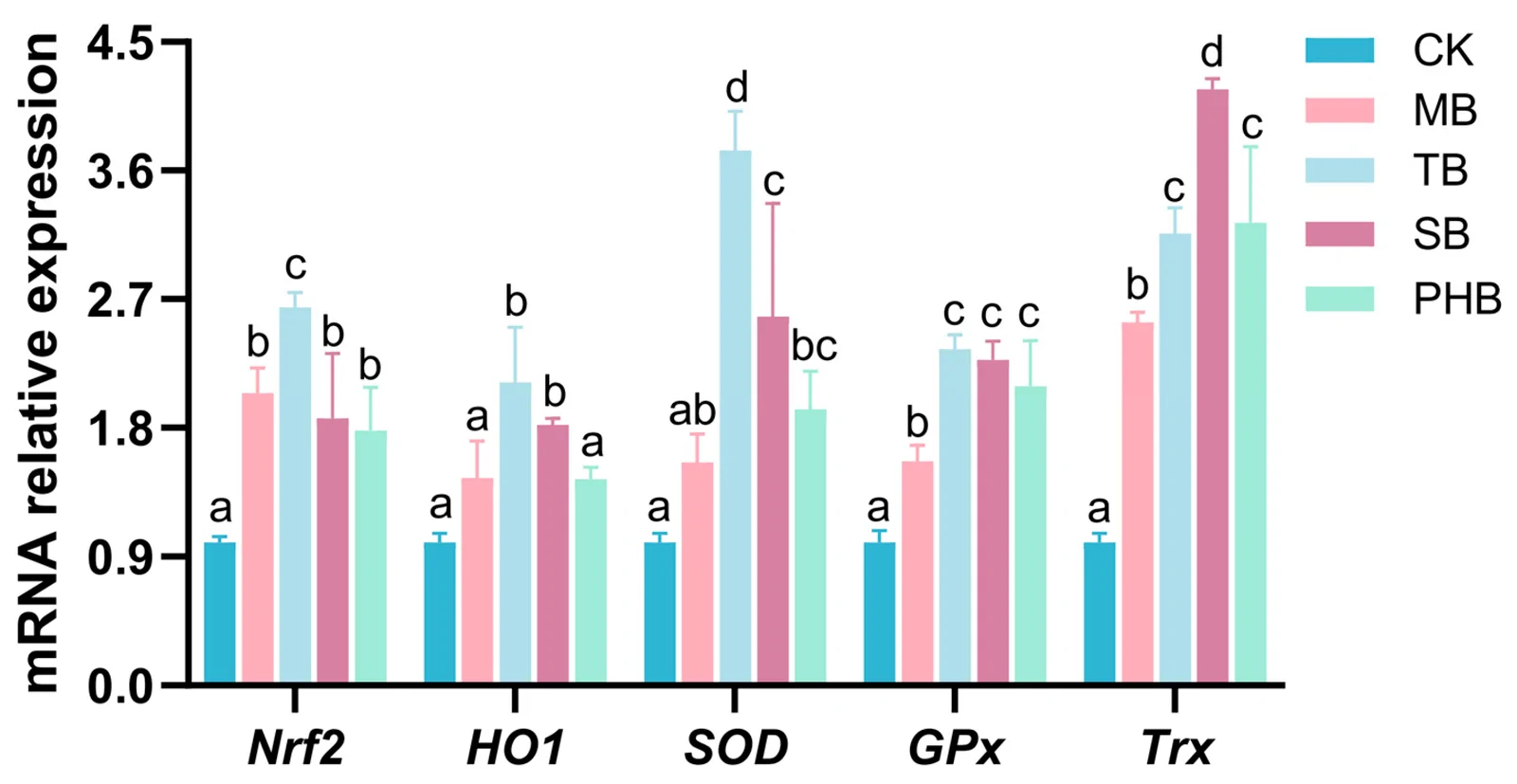
The expression changes in antioxidant-related genes in the intestine of *P. monodon* fed diets supplemented with different types of butyrates for 56 days. Different lowercase letters above error bars indicate significant differences among groups (*p* < 0.05).

The changes in the expression patterns of nuclear factor erythroid-derived 2-like 2 (*Nrf2*) signaling related genes in the shrimp intestines were investigated after dietary supplementation with different types of butyrates (Figure 4). Compared with the CK group, the relative expressions of *Nrf2*, glutathione peroxidase (GPx), and thioredoxin (*Trx*) genes exhibited notable upregulation in the four supplemented groups (*p* < 0.05). Wherein, *Nrf2* expression was highest in the TB group, *GPx* expression peaked in the TB, SB, and PHB groups, while *Trx* expression was highest in the SB group. In addition, the expression of heme oxygenase 1 (*HO1*) gene exhibited notable upregulation in the TB and SB groups (*p* < 0.05), while it rose slightly without statistical significance in the MB and PHB groups (*p* > 0.05). The expression of Cu-Zn superoxide dismutase (*SOD*) gene was markedly elevated in the TB, SB and PHB groups (*p* < 0.05), with the maximum observed in the TB group, whereas only a slight non-significant elevated in the MB group (*p* > 0.05).

### 3.5. Intestinal Immune-Related Gene Expression Changes

**Figure 5:**
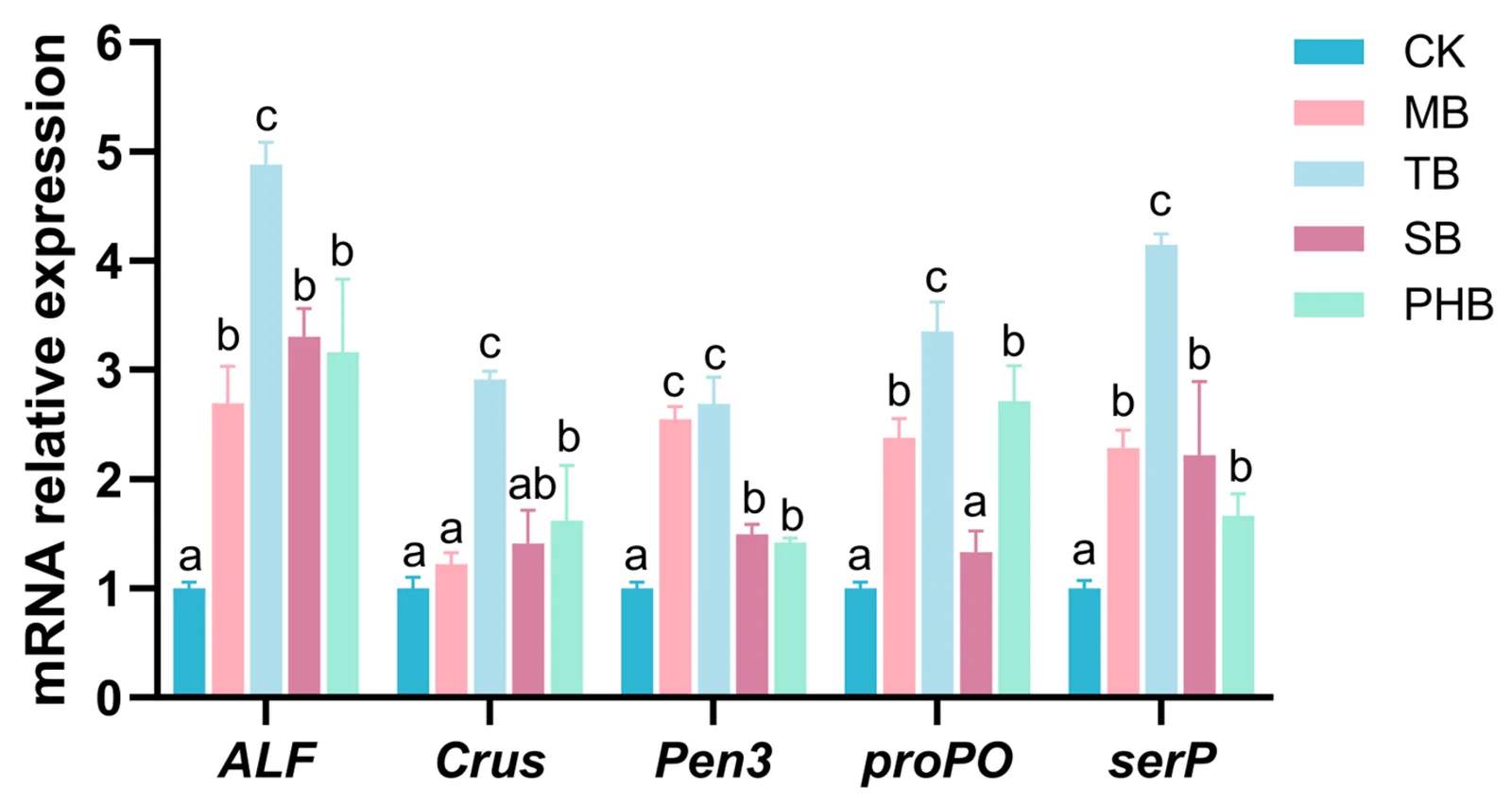
The expression changes in immune-related genes in the intestine of *P. monodon* fed diets supplemented with different types of butyrates for 56 days. Different lowercase letters above error bars indicate significant differences among groups (*p* < 0.05).

The expression patterns of antimicrobial genes in shrimp intestines were determined after dietary supplementation with different types of butyrates (Figure 5). Compared with the CK group, the relative expressions of anti-lipopolysaccharide factor isoform 1 (*ALF*), penaeidin 3a (*Pen3*), and serine protease (*serP*) genes exhibited notable upregulation in the four supplemented groups (*p* < 0.05). Wherein, the expression of *ALF* and *serP* genes reached the maximum in the TB group; the expression of *Pen3* gene was highest in the MB and TB groups. In addition, the expression of crustin (*Crus*) gene exhibited notable upregulation in the TB and PHB groups (*p* < 0.05), while it rose slightly without statistical significance in the MB and SB groups (*p* > 0.05). The expression of prophenoloxidase (*proPO*) gene was markedly elevated in the MB, TB and PHB groups (*p* < 0.05), and peaked in the TB group, whereas only a slight non-significant increase was detected in the SB group (*p* > 0.05).

### 3.6. Intestinal Microbiota Changes

#### 3.6.1. Changes in the Microbial Diversity

**Figure 6:**
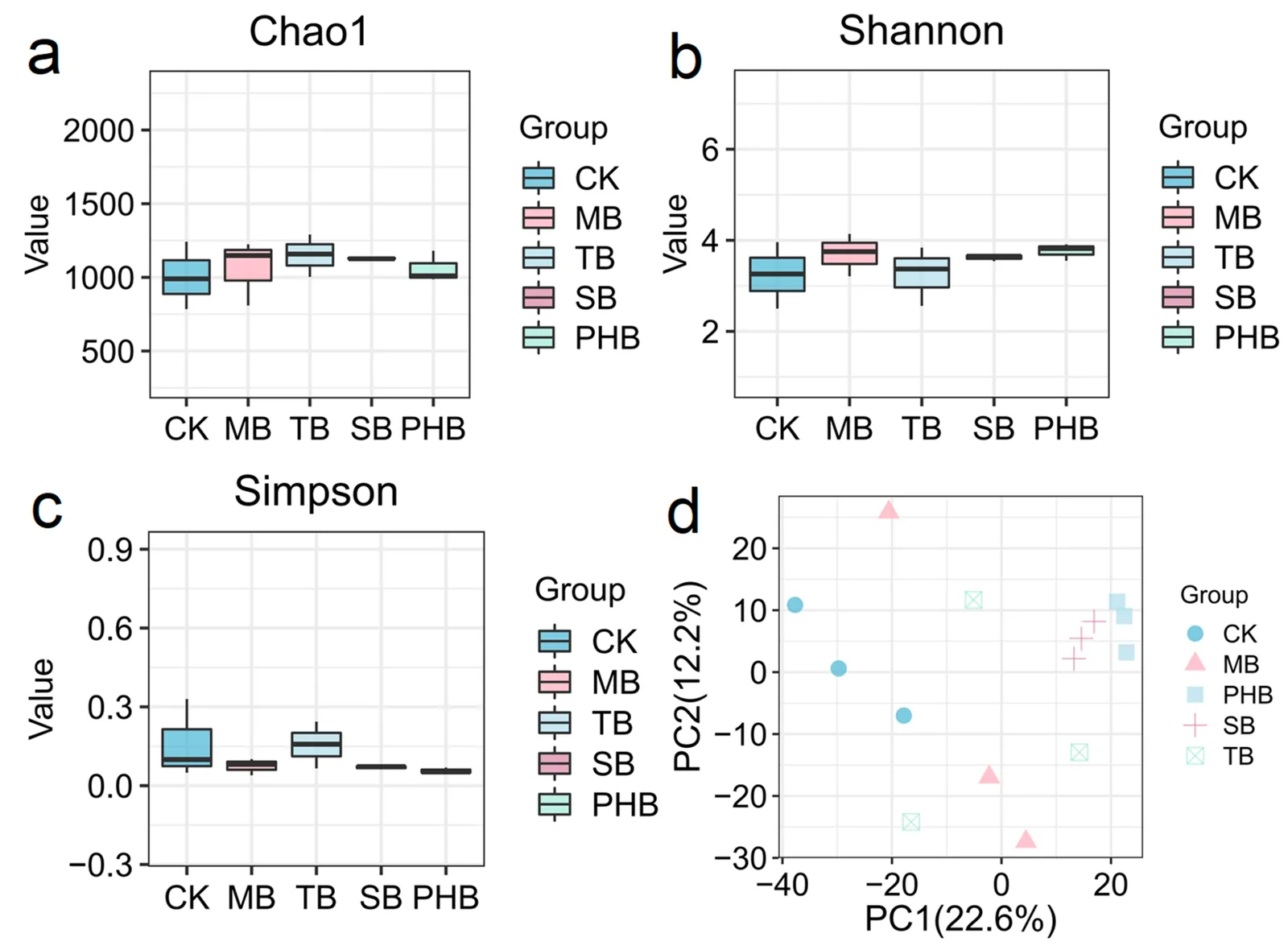
The changes in intestinal microbial diversity of *P. monodon* fed diets supplemented with different types of butyrates for 56 days. (**a**) Chao1 index; (**b**) Shannon index; (**c**) Simpson index; (**d**) The *β*-diversity of intestinal microbiota based on PCA plot.

High-throughput 16S rDNA sequencing was used to characterize intestinal microbial community variations. Each intestinal microbial sample yielded an average of 47,689 valid clean reads. Venn analysis targeting OTU features revealed 488 common OTUs shared by all five experimental groups. Relative to the CK group, the number of unique OTUs decreased sequentially in the MB, SB, TB and PHB groups (Figure S1). For alpha diversity metrics, in relative to the CK group, the Chao1 was higher in the MB, TB, SB and PHB groups; the Shannon index increased in the MB, SB and PHB groups, while no obvious variation in the TB group; and the variation trend of the Simpson index was completely opposite to that of the Shannon index (Figure 6a–c). As shown in the PCA plot, the microbiota profiles of the four supplemented groups clustered separately from the CK group. The PHB and SB groups exhibited a larger separation from the CK, whereas the MB and TB groups were comparatively closer (Figure 6d).

#### 3.6.2. Changes in the Microbial Composition

**Figure 7:**
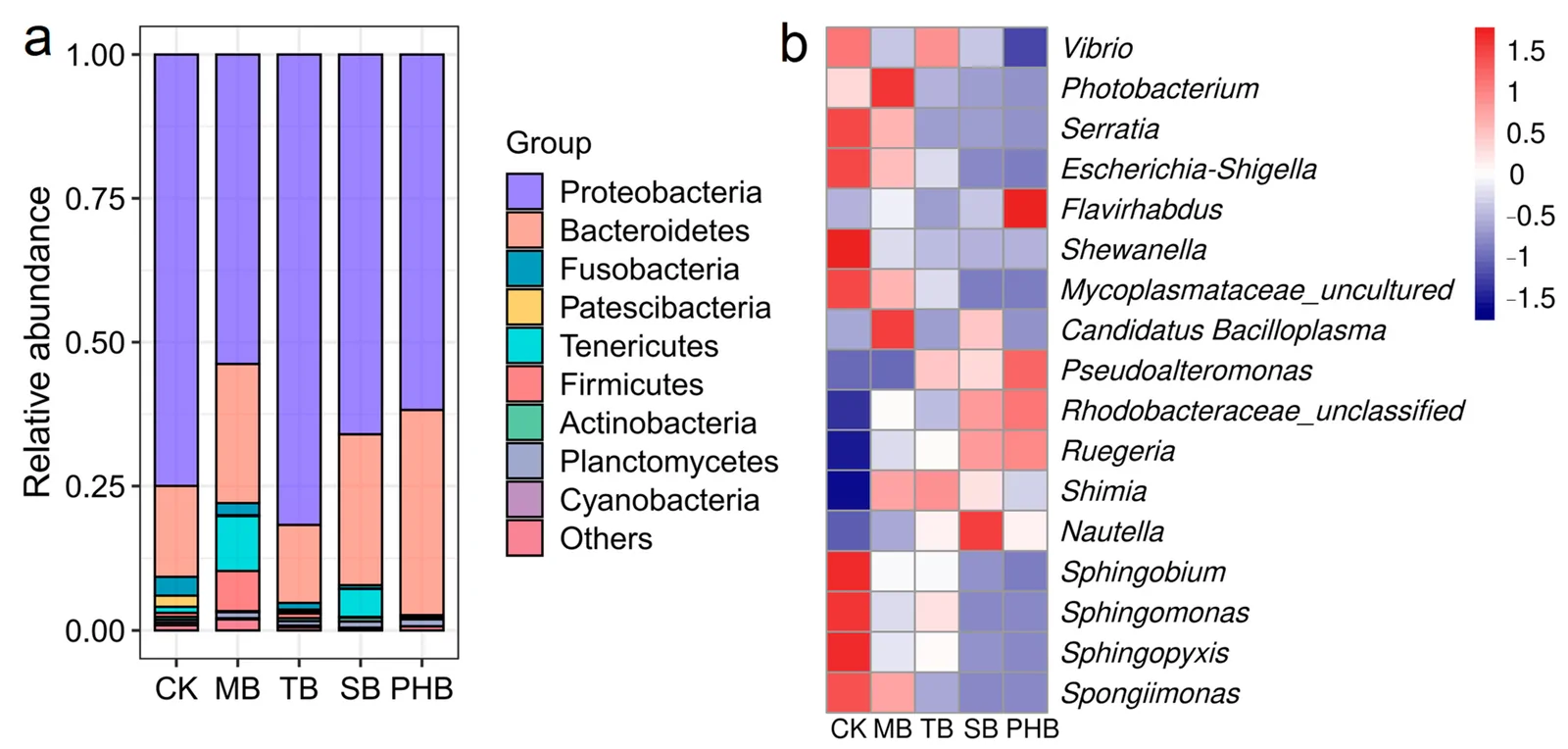
The changes in community composition of intestinal microbiota of *P. monodon* fed diets supplemented with different types of butyrates for 56 days. (**a**) Relative abundance of representative bacterial phyla; (**b**) relative abundance of representative bacterial genera. The color scale ranging from −1.5 to 1.5 corresponds to the z-score of relative abundance, with blue representing decreased abundance and red representing increased abundance.

The intestinal microbial composition was analyzed at multiple taxonomic ranks. In comparison to the CK group, the relative abundances of predominant phyla including Proteobacteria was increased in the TB group, but decreased in the MB, SB and PHB groups; the changing trend of Bacteroidetes was completely opposite to that of Proteobacteria; Fusobacteria and Patescibacteria were decreased in the MB, TB, SB and PHB groups; Tenericutes were increased in the MB and SB groups, but declined in the TB and PHB groups; and Firmicutes rose in the MB group yet declined in the SB and PHB groups (Figure 7a).

Furthermore, the relative abundance of several bacterial genera also changed (Figure 7b and Figure S2). For example, *Ruegeria*, *Shimia*, *Nautella*, and *Rhodobacteraceae_unclassified* rose in the MB, TB, SB and PHB groups, but *Vibrio*, *Serratia*, *Escherichia-Shigella*, *Shewanella*, *Mycoplasmataceae_uncultured*, *Sphingobium*, *Sphingomonas*, *Sphingopyxis*, and *Spongiimonas* declined. *Photobacterium* rose in the MB group, but declined in the TB, SB and PHB groups. *Flavirhabdus* rose in the MB, SB and PHB groups, but declined in the TB group. *Candidatus Bacilloplasma* rose in the MB and SB groups, but declined in the TB and PHB groups. *Pseudoalteromonas* rose in the TB, SB and PHB groups.

#### 3.6.3. Changes in the Microbiota Phenotypes

**Figure 8:**
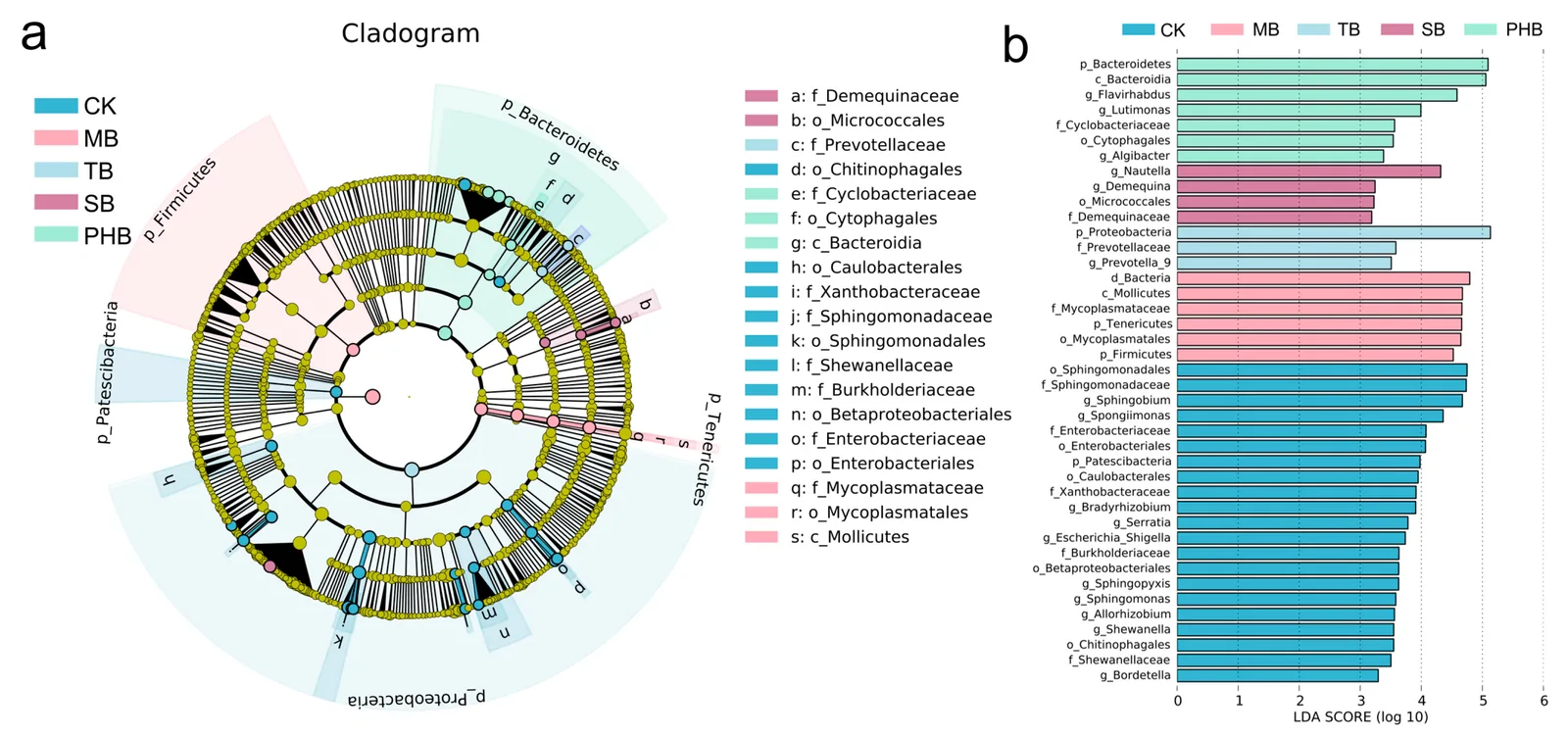
The inter-group variation in intestinal microbiota of *P. monodon* fed diets supplemented with different types of butyrates for 56 days. (**a**) Lefse cladogram; (**b**) LDA score of Lefse-PICRUSt was set with a threshold greater than 3.0.

The group-specific microbial taxa of each group were identified using the LEfSe method. According to cladogram profiling, at the family taxonomic level, Xanthobacteraceae, Sphingomonadaceae, Shewanellaceae, Burkholderiaceae, and Enterobacteriaceae were predominated in the CK group; Mycoplasmataceae was predominated in the MB group; Prevotellaceae was predominated in the TB group; Demequinaceae was predominated in the SB group; and Cyclobacteriaceae was predominated in the PHB group (Figure 8a). Furthermore, among the taxa exceeding an LDA score of 3.0, the genera *Sphingobium*, *Spongiimonas*, *Sphingomonas*, *Sphingopyxis*, *Escherichia_Shigella*, *Bradyrhizobium*, and *Serratia* were predominated in the CK group; *Prevotella 9* was predominated in the TB group; *Nautella* and *Demequina* were predominated in the SB group; and *Algibacter*, *Lutimonas*, and *Flavirhabdus* were predominated in the PHB group (Figure 8b).

#### 3.6.4. Changes in the Microbial Metabolic Functions

**Figure 9:**
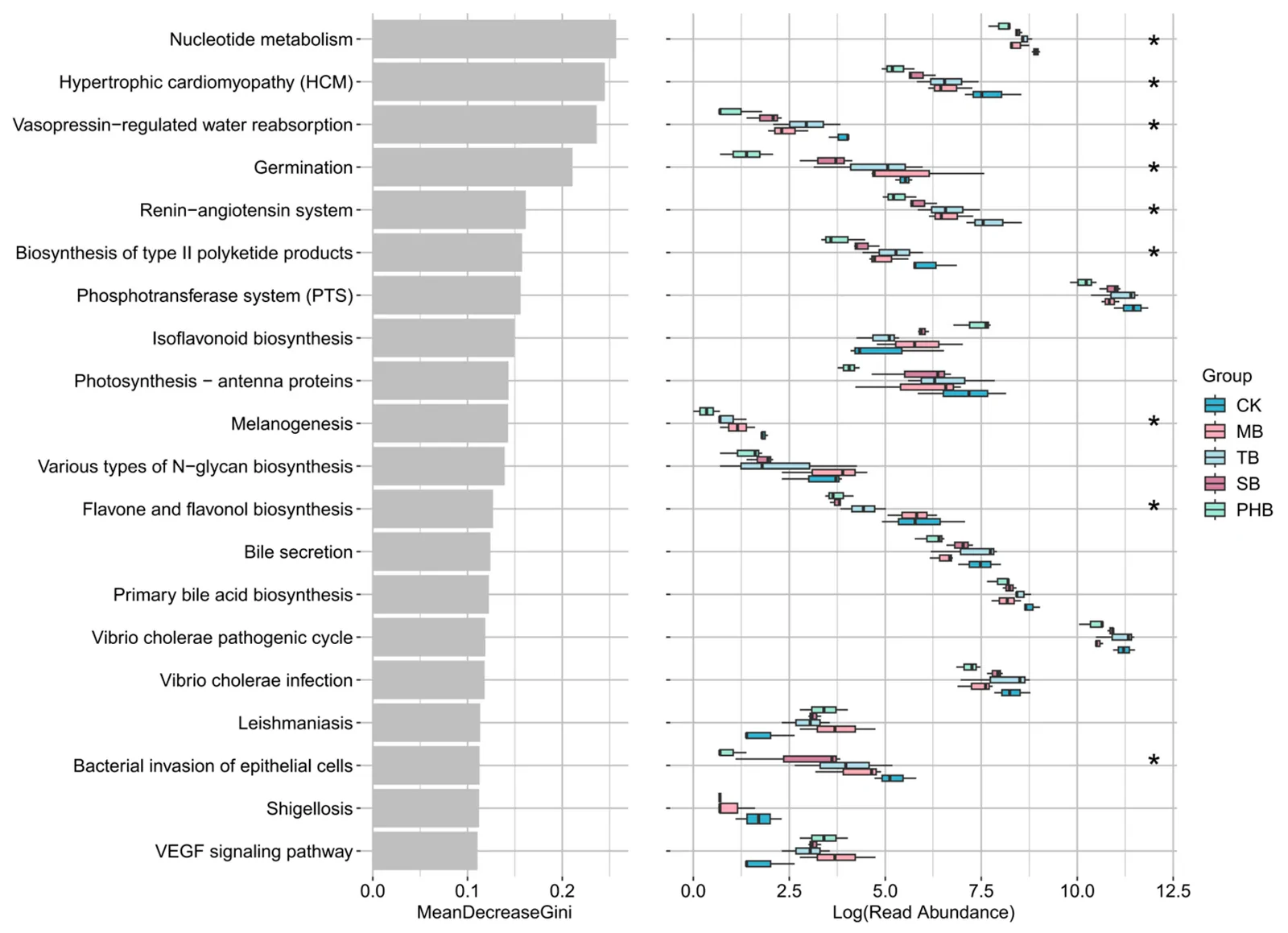
The predicted functional changes in intestinal microbiota of *P. monodon* fed diets supplemented with different types of butyrates for 56 days. * indicates significant difference (*p* < 0.05).

The functional potential of intestinal microbes were further investigated (Figure 9). According to KEGG pathway annotation, relative to the CK group, the functions of “bacterial invasion of epithelial cells”, “biosynthesis of type ii polyketide products”, and “nucleotide metabolism” were declined the MB, TB, SB and PHB groups; “flavone and flavonol biosynthesis” and “germination” were declined the TB, SB and PHB groups.

## 4. Discussion

In shrimp farming systems, the frequent elevation and long-term accumulation of nitrite are major challenges troubling the industry. Nitrite stress induces stress responses in shrimp, weakens their immunity, and easily leads to disease outbreaks [2,3]. Besides regulating water quality, maintaining intestinal homeostasis is also an important strategy to improve shrimp growth performance and stress resistance. Butyrate, as one of the main SCFAs produced by intestinal microbiota metabolism, is widely recognized as a functional substance regulating intestinal health in aquatic animals [15]. Currently, there are various types of butyrate, but whether their roles in regulating intestinal health of shrimp differ remains unclear. On this basis, we explored the impacts of four distinct dietary butyrate additives on the growth, intestinal health, and nitrite stress defense ability of *P. monodon*.

Previous studies have verified that butyrate exerts growth-promoting effects on aquatic organisms [18,23,29]. Consistent with those previous findings, after 56 days of feeding diets containing various butyrate derivatives, shrimp in all supplementation groups exhibited markedly elevated weight gain alongside a notable reduction in feed conversion ratio. Moreover, the shrimp survival was significantly improved under both normal culture conditions and after 48 h of nitrite stress. Among the four butyrate types, TB and PHB exhibited the most prominent promoting effects, followed by MB and SB. These results indicated that butyrate could effectively enhance the growth performance and feed efficiency of *P. monodon*, as well as enhance their stress resistance against nitrite, and that these effects were closely related to the specific type of butyrate.

Intestinal mucosal integrity and antioxidant capacity are important indicators for assessing intestinal health status [4]. In the present study, after 56 days of dietary supplementation with different butyrates, the intestinal mucosal morphology of *P. monodon* was improved, which was beneficial to the maintenance of intestinal physical barrier function and physiological homeostasis. Redox homeostasis is an important indicator for evaluating intestinal health. MDA and LPO can reflect oxidative stress damage, while the antioxidant enzyme system can defend against oxidative stress [37,38]. Nrf2, as a pivotal transcription factor, upregulates a battery of antioxidant effectors, including *GPx*, *SOD*, *HO1*, and *Trx*, consequently alleviating oxidative stress [39,40]. In the present study, after 56 days of feeding with butyrate-supplemented diets, the intestinal contents of MDA and LPO in *P. monodon* were decreased, while the activities of T-AOC, SOD, CAT, and ASC generally showed an increasing trend. Meanwhile, the *Nrf2*, *GPx*, and *Trx* genes were significantly induced in all four butyrate groups, and the expression of *HO1* and *SOD* was also significantly upregulated in the TB and SB groups. These biochemical and molecular changes corroborated each other, indicating that butyrate could attenuate lipid peroxidation damage and enhance antioxidant defense capacity through the activation of the intestinal *Nrf2* pathway and its downstream antioxidant genes. However, the regulatory effects varied among different butyrate types, with TB and SB exhibiting superior performance in inducing key antioxidant effectors, while the effects of MB and PHB were relatively less pronounced.

The shrimp intestine acts as the primary barrier resisting pathogenic invasion, and its immune homeostasis is vital to maintaining overall health [9]. As important components of the shrimp immune system, ALF, Crus, and Pen3 are three types of antimicrobial peptides [41]; serP can activate the proPO system to initiate melanization and pathogen clearance [42]. In the present study, all four dietary butyrate types could upregulate the expression of immune-related genes *ALF*, *Pen3*, and *serP* in the intestine of *P. monodon*, whereas *Crus* and *proPO* were significantly upregulated only in certain treatment groups, with TB and PHB showing the most prominent effects. These results indicated that dietary butyrate supplementation could activate intestinal immune responses in shrimp, but the immunomodulatory efficacy varied considerably among different butyrate derivatives. TB and PHB exhibited more pronounced improvements in intestinal immunity, likely due to their long-term sustained-release properties, which were better adapted to the intestinal physiological environment of shrimp. MB showed moderate immunomodulatory effects, probably because of its limited sustained-release capacity and the fact that it released only one molecule of butyrate. SB exhibited the weakest overall regulatory effects, which might be attributed to its water-soluble nature, leading to rapid butyrate release in the gastrointestinal tract.

Intestinal microbiota plays a key role as a biological barrier in regulating host nutrient metabolism, immunity, and defense against exogenous invasion [43]. Gut microbial diversity exhibits tight correlations with the physiological status of shrimp [11]. In the present study, although the diversity indices of intestinal microbiota did not change significantly after shrimp were fed with four types of butyrate, they showed an overall increasing trend; this contributes to strengthening the stability and anti-interference capacity of the intestinal microecosystem. Furthermore, the composition of intestinal microbiota was also regulated by dietary butyrate. Bacteroidetes and Proteobacteria represent the two most abundant bacterial phyla residing within the digestive tract of aquatic species. Bacteroidetes mainly participates in the metabolism of host carbohydrates and proteins [44], while Proteobacteria also possess broad metabolic capabilities [45]. In the present study, Proteobacteria levels rose in the TB group but fell in the MB, SB, and PHB groups, with Bacteroidetes following a completely opposite trajectory to that of Proteobacteria. This phenomenon indicated that dietary butyrate might influence host metabolism by reshaping the ecological niche of dominant bacteria, and also demonstrated that the regulatory effects of butyrate on dominant bacterial phyla are type-dependent.

Dietary butyrate additives also reshaped the balance between potentially beneficial and opportunistic pathogenic bacteria in the shrimp intestine. *Vibrio* is a common opportunistic pathogen in shrimp farming [46]; *Serratia*, *Escherichia-Shigella*, and *Shewanella* are also considered opportunistic pathogens [47,48]. In the present study, *Vibrio*, *Serratia*, *Escherichia-Shigella*, and *Shewanella* abundances were decreased in all of the four supplementation groups, indicating that dietary butyrate was beneficial for reducing the risk of disease outbreaks. Rhodobacteraceae is a common bacterial family in the intestinal of marine animals and in aquaculture environments, and possesses metabolic capacity [49]. In the present study, Rhodobacteraceae bacteria such as *Ruegeria*, *Shimia*, *Nautella*, and *Rhodobacteraceae_unclassified* were increased in all four supplementation groups, suggesting that dietary butyrate might enhance the metabolic capacity of the intestinal microbiota. Sphingomonads, such as *Sphingomonas*, *Sphingobium*, and *Sphingopyxis*, possess xenobiotic-degrading capabilities, and play pivotal roles in adapting to oligotrophic niches and degrading refractory polycyclic aromatic hydrocarbons [50]. In the present study, these three Sphingomonadaceae genera abundances were decreased in all four supplementation groups, indicating that dietary butyrate might remodel the intestinal microecology, causing these pollutant-degrading bacteria to no longer dominate, which indirectly reflected changes in the intestinal environment. *Photobacterium* is pathogenic to shrimp [51], while *Pseudoalteromonas* has been reported as a probiotic [52]. In the present study, *Photobacterium* abundance only rose in the MB group, but declined in the TB, SB, and PHB groups; *Pseudoalteromonas* displayed elevated levels across the TB, SB, and PHB groups. These findings indicated that dietary butyrate could selectively inhibit potential pathogens in the intestine while promoting the proliferation of certain beneficial bacteria, thereby reshaping an intestinal microecology that was more favorable to host health. It was worth noting that type-dependent characteristic changes in intestinal microbiota regulation also exist among different butyrate forms.

Furthermore, the abundance of the bacterial invasion of epithelial cells pathway in the intestinal microbiota of shrimp in the four butyrate-supplemented groups was decreased, which was logically consistent with the improvement in intestinal mucosal structure. Combined with the results of intestinal histological observation, dietary butyrate supplementation improved the intestinal tissue integrity of *P. monodon*. An intact intestinal physical barrier can hinder the adhesion and invasion of pathogenic bacteria, providing corroborative evidence for the decreased abundance of the bacterial invasion pathway at the microbiota level. Notably, this association was only a qualitative trend analysis and did not represent a quantitative correlation, which still requires further in-depth investigation in future studies.

## 5. Conclusions

**Figure 10:**
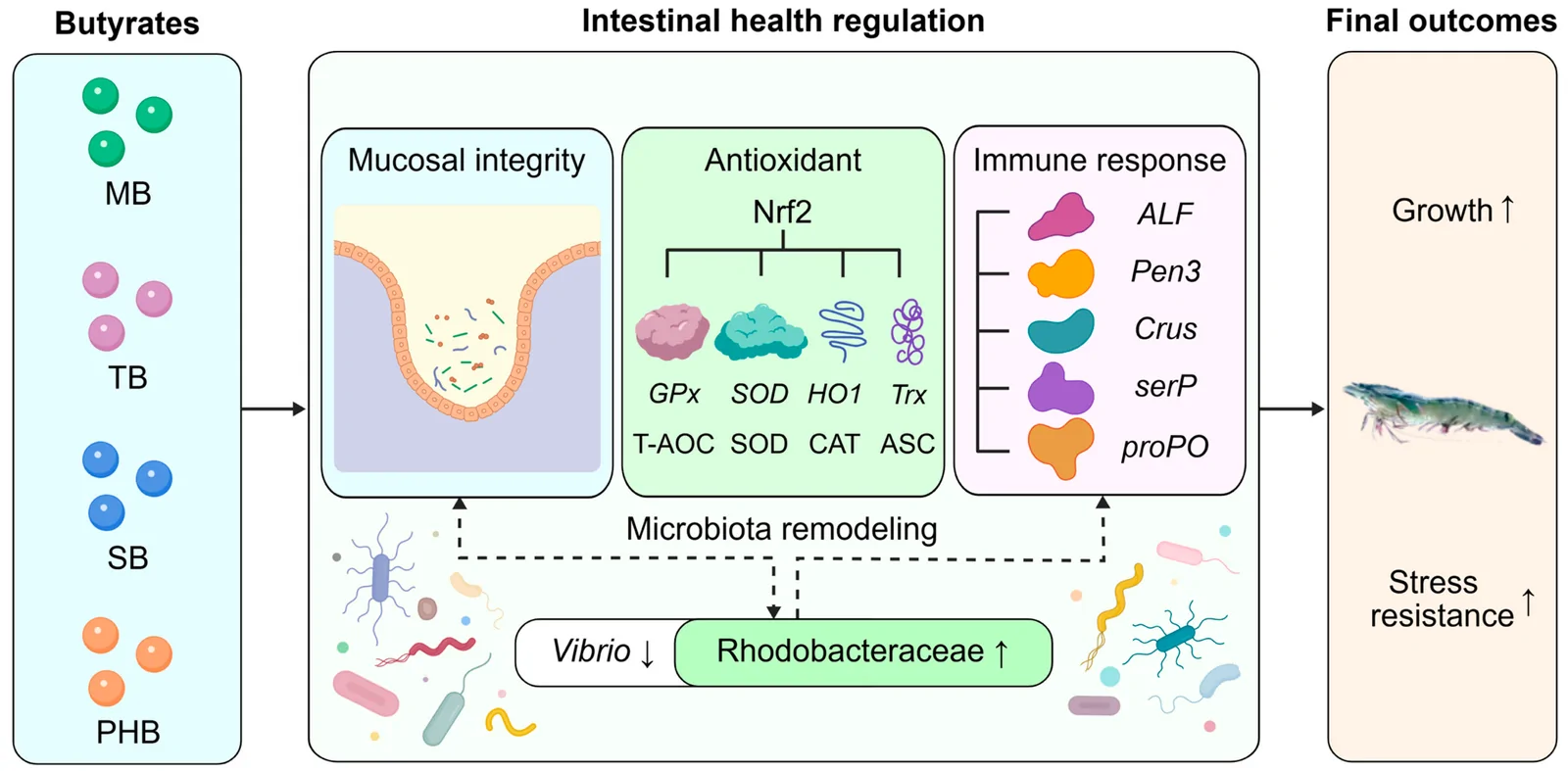
Derivation of the effects of four types of butyrate on growth, intestinal health, and nitrite resistance of *P. monodon*.

This study systematically compared and investigated the effects of four butyrate types (MB, TB, SB, and PHB) on the growth, intestinal health, and nitrite stress tolerance of *P. monodon*. Specifically, all four butyrate types improved intestinal health to varying degrees by maintaining intestinal mucosal integrity and enhancing antioxidant and immune capacities, thereby promoting growth and enhancing nitrite stress resistance in *P. monodon* (Figure 10). However, different molecular configurations of butyrate exerted differential regulatory effects on the health of *P. monodon*. Among them, under the conditions of this study, TB exhibited the optimal effect, followed by PHB, MB and SB. From the perspective of industrial application in aquafeed, TB and PHB can be prioritized as butyrate-based additives for *P. monodon* feed. These findings provide a practical basis for the development and scientific application of different butyrate configurations as feed additives in shrimp aquaculture.

## Figures and Tables

**Table 1:** Growth performance of *P. monodon* fed diets supplemented with different types of butyrates for 56 days.

Indicators	CK	MB	TB	SB	PHB
Initial weight (g)	2.08 ± 0.01 ^a^	2.10 ± 0.01 ^a^	2.11 ± 0.02 ^a^	2.10 ± 0.01 ^a^	2.09 ± 0.02 ^a^
Final weight (g)	7.00± 0.131 ^a^	8.33± 0.18 ^b^	9.30 ± 0.16 ^c^	8.50 ± 0.16 ^b^	9.11 ± 0.17 ^c^
Weight gain (%)	235.80 ± 5.69 ^a^	296.98 ± 11.51 ^b^	341.79 ± 8.72 ^c^	305.97 ± 9.45 ^b^	336.07 ± 8.01 ^c^
Feed conversion rate (FCR)	1.68 ± 0.05 ^b^	1.36 ± 0.03 ^a^	1.32 ± 0.04 ^a^	1.39 ± 0.08 ^a^	1.30 ± 0.05 ^a^

The values marked with different lowercase letters indicate significant differences among groups (*p* < 0.05).

## Data Availability

The raw data supporting the conclusions of this article will be made available by the authors on request.

## Supplementary Materials

The following supporting information can be downloaded at: https://www.mdpi.com/article/10.3390/antiox15080929/s1, Table S1: Formulation and proximate composition of experimental diets. Table S2: The qPCR primer sequences used in this study. Figure S1: Venn diagram of the OTUs of intestinal microbiota of *P. monodon* fed diets supplemented with different types of butyrates for 56 days. Figure S2: Heatmap of relative abundance of intestinal bacterial genera of *P. monodon* fed diets supplemented with different types of butyrates for 56 days.
