# Supplementary material for: Comparative Efficacy of Monobutyrin, Tributyrin, Sodium Butyrate, and Poly-β-hydroxybutyrate on Growth, Intestinal Health, and Nitrite Stress Resistance in Penaeus monodon
Source: Antioxidants (Basel). 2026 Jul 27;15(8):929. doi: 10.3390/antiox15080929 (PMC13509874; doi:10.3390/antiox15080929)
Supplement: Supplementary file 1 [file antioxidants-15-00929-s001.zip › antioxidants-4426675-supplementary.pdf]

Table S1

Formulation and proximate composition of experimental diets.

Group	CK	MB	TB	SB	PHB
Ingredients (% dry matter)					
White fish meal[a]	31	31	31	31	31
Soybean meal[b]	16.3	16.3	16.3	16.3	16.3
Peanut bran[b]	17	17	17	17	17
Wheat flour[b]	19	18	18	18	18
Beer yeast[b]	5	5	5	5	5
Krill meal[b]	3	3	3	3	3
Soybean lecithin[c]	2	2	2	2	2
Fish oil[d]	1	1	1	1	1
Soybean oil[b]	1	1	1	1	1
Choline chloride (50%)	0.6	0.6	0.6	0.6	0.6
$Ca(H_2PO_4)_2$	1	1	1	1	1
Squid meal paste	1	1	1	1	1
V_C-phosphate ester[e]	0.1	0.1	0.1	0.1	0.1
Vitamin premix[f]	0.5	0.5	0.5	0.5	0.5
Mineral premix[g]	0.5	0.5	0.5	0.5	0.5
Sodium alginate	1	1	1	1	1
Butyrate additive	0	1	1	1	1
Total	100	100	100	100	100
Nutrient value (% dry weight)					
Crude protein	40.13	40.08	39.96	40.05	39.91
Lipid	8.93	8.95	8.97	8.91	8.92
Ash	10.41	10.45	10.48	10.42	10.46
Moisture	10.12	10.13	10.08	10.02	10.17

[a] Imported from N.E.L.T.O. Australia Pty Ltd.

[b] Zhuhai Shihai Feed Corporation Ltd., Zhuhai, China.

[c] Kemin Industries (Zhuhai) Ltd., Zhuhai, China.

[d] Imported from New Zealand (Bakels Edible Oils Ltd, Mt Macnganui).

[e] Guangzhou Chengyi Company Ltd., Guangzhou, China.

[f] Vitamin premix ($g\ kg^{-1}$): retinyl acetate, 2.5; cholecalciferol, 6.25; all-rac-atocopheryl acetate, 75; menadione, 2.5; thiamin, 0.25; riboflavin, 1; D-calcium pantothenate, 5; pyridoxine HCl, 0.75; cyanocobalamin, 2.5; niacin, 2.5; folic acid 0.25; biotin 2.5; meso-inositol, 379; cellulose, 500.

[g] Mineral premix ($g\ kg^{-1}$): KCl, 90; KI, 0.04; NaCl, 40; $CuSO_4$-$5H_2O$, 3; $ZnSO_4$-$7H_2O$, 4; $CoSO_4$-$7H_2O$, 0.02; $FeSO_4$-$7H_2O$, 20; $MnSO_4$-H_2O, 3; $MgSO_4$-$7H_2O$, 124; $CaHPO_4$-$2H_2O$, 500; $CaCO_3$, 215.

Table S2

The PCR primer sequences used in this study.

Primer name	Sequence (5'-3')
Nrf2-F	CATCAGGTCTGTCACGTCCAACTTC
Nrf2-R	CTGGCTCGCTTCTCATCTCTTGTG
HO1-F	AAGGAGCCGGTAGTGTTGTG
HO1-R	AGCCTGTACATTTGCCCCTC
GPx-F	CTGGAGGCAGTGGCTTAGAAGTTG
GPx-R	TCAGCAGAGGGAAGTCAGGAAGG
SOD-F	GAAACCATCCTCGTCCACACACTC
SOD-R	GCGACGTAGGCGATGCACTTC
Trx-F	TGGAAACAAGCTGGTCGTCATCG
Trx-R	CATTCGTCCACATCCACCTTCAGG
ALF-F	CGTATTTGACTTTGCGGTGCTTCC
ALF-R	GCGTCTTCCTCCGTGATGAGATTAC
Crus-F	TGTCCAAGGAGGCGGCTTAGG
Crus-R	ACCACCCAAACCACCACTTAAACC
Pen3-F	TCCCAGCAGGTCCTCGTGTATTC
Pen3-R	CACCAACCACACACAGACCCATAC
proPO-F	ATTTAACTTCTGCGGGTGTGGATGG
proPO-R	GTCCTGGGCGTAGTCGGTGAG
serP-F	GCGGTGCTAAGGACGGAATACTC
serP-R	TTGAGGGTGGTTGGGTTGATGTTG
β-actin-F	GCAAGCCACAGCCACGAGAAG
β-actin-R	CACATGCCGGAGCCATTGTCTAC
515F	GTGCCAGCMGCCGCGG
806R	GGACTACHVGGGTWTCTAAT

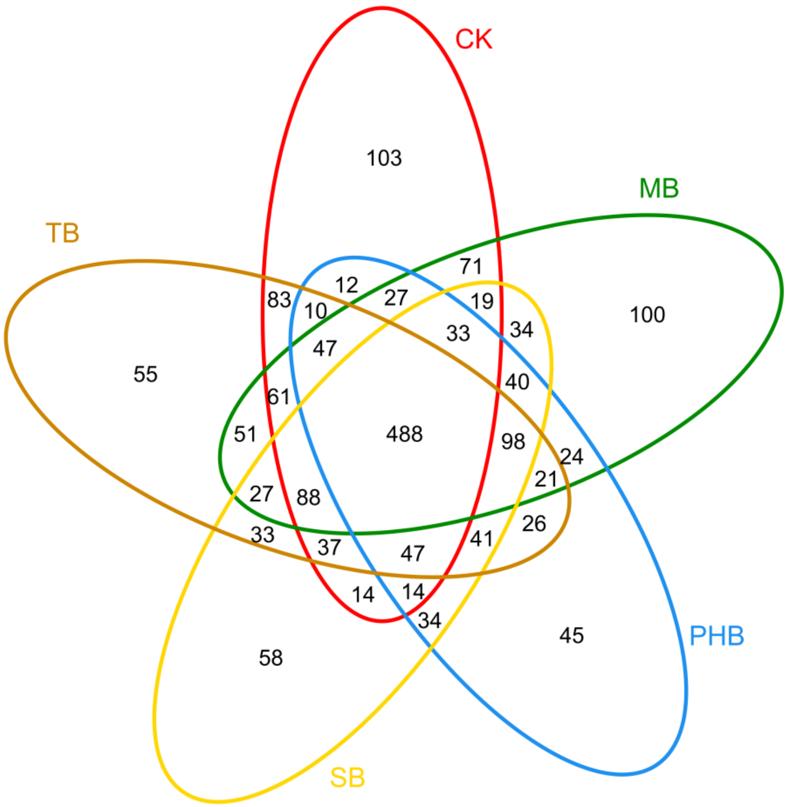

Figure S1. Venn diagram of the OTUs of intestinal microbiota of *P. monodon* fed diets supplemented with different types of butyrates for 56 days.

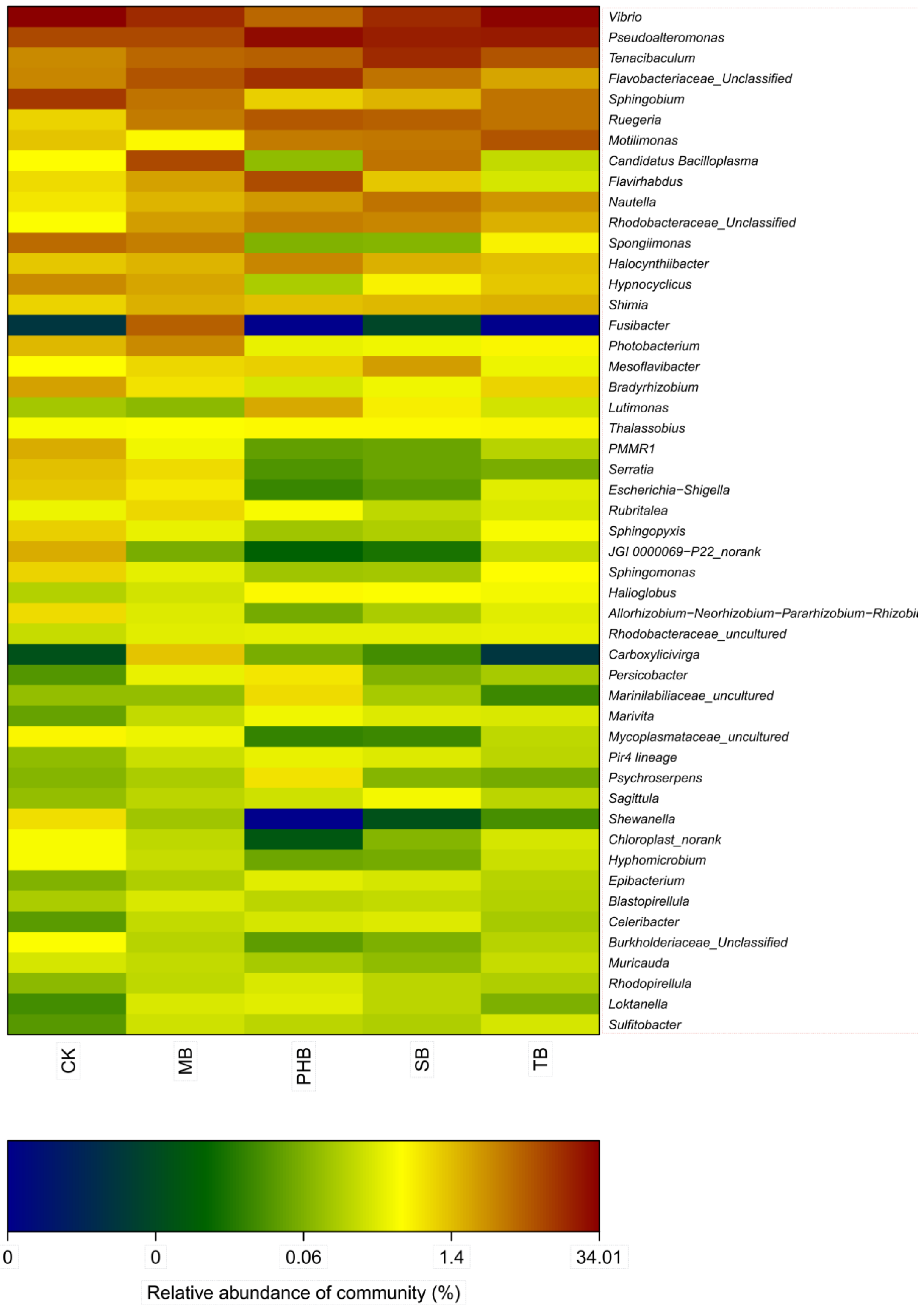

Figure S2. Heatmap of relative abundance of intestinal bacterial genera of *P. monodon* fed diets supplemented with different types of butyrates for 56 days.

The detailed experimental methods

2.6. Histomorphological Analysis

2.6.1 Tissue fixation

Fresh intestinal tissue samples were fixed in 4% paraformaldehyde solution for 24 h.

2.6.2 Embedding and sectioning

① Washing: Samples were rinsed with running tap water for 30 min.

② Dehydration: After washing, tissue blocks were immersed in 70% ethanol overnight. On the next day, samples were sequentially soaked in 80%, 90% and 100% ethanol for 1 h each.

③ Clearing: Samples were treated with acetone, xylene A and xylene B for 30 min each until the tissue blocks turned tan or dark red and transparent.

④ Wax infiltration: Tissue blocks were transferred to paraffin baths A, B and C in a water bath incubator at 60 °C for 1 h per bath.

⑤ Embedding: Tissue blocks were placed in embedding molds and embedded using an embedding machine.

⑥ Sectioning: Sections with a thickness of 4 μm were prepared with a microtome. The obtained tissue sections were dried in an oven at 45 °C overnight.

2.6.3 Hematoxylin–Eosin (HE) staining

① Deparaffinization: Paraffin sections were placed in staining containers and deparaffinized in xylene A and xylene B for 10 min each. The deparaffinization time could be extended appropriately to completely remove paraffin from tissues.

② Gradient ethanol rehydration: Sections were sequentially immersed in 100%, 95%, 90%, 85% and 70% ethanol for 5 min each. After rehydration, sections were washed twice with distilled water for 5 min each time.

③ Nuclei staining with hematoxylin was carried out for 5 min; followed by rinsing under running tap water for 1 min and standing in still water for 5 min.

④ Staining with 0.5% eosin solution was carried out for 1 min, then samples were thoroughly rinsed with running tap water. Slides were air-dried in a fume hood.

⑤ Mounting: Dried tissue sections were mounted with neutral balsam.

2.6.4 Image acquisition

Images were captured under an optical microscope (Nikon, Tokyo, Japan).

2.7. Biochemical Analysis

Intestinal tissues were homogenized by adding sterile physiological saline solution to prepare 10% (W:V) homogenates. Homogenates were centrifuged at 4 °C and 3500 rpm for 15 min. After removing precipitates, the supernatants were used for oxidative stress-related biochemical indicators. Among these, malondialdehyde (MDA) and lipid peroxidation (LPO) represent oxidative stress damage, while total antioxidant capacity (T-AOC), total superoxide dismutase (SOD), catalase (CAT), and anti-superoxide anion capacity (ASC) reflect antioxidant capacity. All the indicators were measured using commercial kits from the same batch produced by Nanjing Jiancheng Bioengineering Institute (Nanjing, China), and the final detection was performed on a microplate reader (Spark, Tecan, Austria).

The principle of LPO measurement: Under the reaction condition of 45 °C for 60 min, one molecule of LPO reacts with two molecules of chromogenic reagent to generate a stable chromophore, which has a maximum absorption peak at 586 nm. The LPO content in the test sample can be obtained by comparison with the standard curve or calculated through a formula.

MDA measurement adopts the thiobarbituric acid (TBA) method. Its principle is that MDA can condense with TBA to form a red product, which has a maximum absorption peak at 532 nm.

T-AOC measurement uses the colorimetric method. Its principle is that there are many antioxidant substances in the organism that can reduce Fe^{3+} to Fe^{2+}. The latter can form stable complexes with phenanthroline substances, and the level of antioxidant capacity can be measured by colorimetry.

CAT measurement adopts the ammonium molybdate method. Its principle is that the reaction in which catalase decomposes H_2O_2 can be quickly terminated by adding ammonium molybdate. The remaining H_2O_2 reacts with ammonium molybdate to form a pale yellow complex. Its variation is measured at 405 nm to calculate CAT activity.

SOD measurement adopts the hydroxylamine method. Its definition: The amount of SOD corresponding to a 50% inhibition rate of SOD in 1 mL of reaction solution per milligram of tissue protein is one unit (U) of SOD activity.

ASC measurement adopts the colorimetric method. Its definition: In the reaction system, the variation value of superoxide anion radicals inhibited by 1 g of tissue protein after reacting at 37 °C for 40 minutes, which is equivalent to the superoxide anion radicals inhibited by 1 mg of vitamin C, is one activity unit U.
